# Understanding the Mechanosensitivity of the Median Nerve in Pre-Surgical Carpal Tunnel Syndrome Patients: A Correlational Study

**DOI:** 10.3390/brainsci14060615

**Published:** 2024-06-19

**Authors:** Mar Hernández-Secorún, Hugo Abenia-Benedí, María Orosia Lucha-López, María Durán-Serrano, Javier Sami Hamam-Alcober, John Krauss, Christie Booth-Smith, César Hidalgo-García

**Affiliations:** 1 Unidad de Investigación en Fisioterapia, Faculty of Health Science, Universidad de Zaragoza, 50009 Zaragoza, Spain; marhsecorun@unizar.es (M.H.-S.); hugoabenia1@gmail.com (H.A.-B.); hidalgo@unizar.es (C.H.-G.); 2 Unit of Reconstructive Surgery of the Locomotor System, Hand-Microsurgery, Department of Orthopaedic Surgery and Traumatology, Hospital Universitario Miguel Servet, 50009 Zaragoza, Spain; mdurans@salud.aragon.es (M.D.-S.); jshamam@salud.aragon.es (J.S.H.-A.); 3 School of Health Sciences, Oakland University, Rochester, MI 48309, USA; krauss@oakland.edu (J.K.); cmbooth3@gmail.com (C.B.-S.)

**Keywords:** carpal tunnel syndrome, outcome assessment, health care

## Abstract

(1) Background: Neurodynamic tests are recommended for the diagnosis of entrapment neuropathies such as carpal tunnel syndrome (CTS). However, their association with clinical variables in severe patients or patients with associated comorbidities is poorly documented. This study aims to analyze the association between the mechanosensitivity of the median nerve and symptoms, function and psycho-social variables in moderate and severe carpal tunnel syndrome patients with comorbidities; (2) Methods: Correlational study. In total, 42 pre-surgical patients (24 females; 59.1 ± 12.7 years) included in the Spanish Public Healthcare System with an electrodiagnostic of CTS were selected. Sociodemographic variables and clinical features (symptoms, function, sensitivity and quality of life evaluated with the 36-item Short Form Survey (SF-36) and the Medical Outcomes Study Sleep Scale (MOS-sleep) were recorded. Upper Limb Neurodynamic Test 1 was used to evaluate neural mechanosensitivity; (3) Results: The 81% had a severe CTS and 78.6% had some comorbidity. The average time from the first medical visit to the surgeon’s visit was 365.5 days. Median nerve mechanosensitivity correlated weakly with the SF-36 subscale, General Health, (Spearman’s rho = 0.367) and MOS sleep scale, Awaken Short of Breath or with headache dimension (Spearman’s rho = −0.353) and moderately with SF-36 subscale, Social Functioning (Spearman’s rho = 0.553); (4) Conclusions: No associations were observed for median nerve mechanosensitivity, except for quality of life and sleep. Both social determinants and clinical variables should be considered when examining and treating these patients.

## 1. Introduction

Carpal Tunnel Syndrome (CTS) is the most common peripheral neuropathy in the upper extremity [1,2]. The prevalence has been increasing over the years, from 26.03/10,000 in 1993 to 36.08/10,000 in 2013 in the UK (1:3 for women) [3]. Nerve conduction testing is considered the gold standard for diagnosing CTS [4]. However, it is not universally available, and referral to other health care services for the purpose of diagnosis of CTS increases waiting time and healthcare costs [5]. Furthermore, given the complexity and variability in the use of nerve conduction tests for the diagnosis of CTS, it is necessary to combine them with other diagnostic methods to improve accuracy [6,7,8]. Therefore, a detailed clinical assessment and, in some cases, other imaging modalities, are recommended to obtain a more accurate and complete diagnosis [9]. One of the tests used for the assessment of CTS is neurodynamic testing. The Upper Limb Tension Test 1 (ULNT1) uses a combination of movements of the upper limb to expose the median nerve to 18% of strain, with 4% of the strain at the distal part of the nerve [10]. The Upper Limb Tension Test 1 is considered a valid test for carpal tunnel syndrome [11,12,13,14]. However, it has been observed that these tests do not behave in the same way based on the clinical and psychosocial presentations of the patient. Baselgia et al. [15] observed that patients with a negative ULNT1 still presented with diminished heat detection. However, these authors only focused on the relationship between neurodynamic testing and nociception, without taking into account other aspects such as patient function or their psychosocial status. Furthermore, no literature that associates ULNT1 with the clinical variables used during the assessment of pre-surgical CTS patients was found.

This study aimed to analyze the association between the median nerve’s mechanosensitivity and symptoms, function, and psychosocial variables in pre-surgical carpal tunnel syndrome patients.

## 2. Materials and Methods

### 2.1. Study Design

This study used an observational and analytical cross-sectional design. Patients with CTS included in a surgery waiting list from Healthcare Sector System II of Aragon (Zaragoza, Spain) were referred to the University of Zaragoza. The examination was conducted at the Faculty of Health Sciences at the University of Zaragoza (50009 Zaragoza, Spain) between May 2021 and November 2022 by two researchers (M.H.-S. and H.A.-B.). This study was conducted in accordance with the Declaration of Helsinki and approved by the Local Ethics Committee “Comité Ético de Investigación Clínica en Aragón” (nº05/2021 on 10 March 2021). STROBE statement was used in this study (Table S1).

### 2.2. Sample Size Calculation

The sample size was estimated assuming an infinite population. The expected prevalence of carpal tunnel syndrome used was 14.0% [16]. The GRANMO v.7.12 software (Program of Research in Inflammatory and Cardiovascular Disorders. Institut Municipal d’Investigació Mèdica, Barcelona, Spain. https://www.imim.cat/media/upload/arxius/granmo/granmo_v704.html (accessed on 15 May 2021)) was used to compute the sample size [17], with a 0.95 confidence level and a desired precision of +/− 12 percent units in the population estimation option. A minimum of 33 participants was recommended. To account for the possibility of participant loss or incomplete survey data, it was decided that 42 participants would be recruited.

### 2.3. Participants

Participants were recruited from the surgery waiting list for a carpal tunnel release from Healthcare Sector System II of Aragon (Zaragoza, Spain), composed of two medical specialisation centres and a University Hospital. One researcher from the Unit of Reconstructive Surgery of the Locomotor System (M. D.-S.) oversaw the selection criteria for referring potential patients for inclusion in the study and informing patients about the study via an information leaflet. One researcher from the University of Zaragoza phoned potential individuals who expressed interest in participating in the study.

Inclusion criteria were: (1) >18 years old; (2) currently experiencing CTS diagnosed by a nerve conduction study; (3) inclusion on the surgical waiting list of Sector II of Aragon; (4) more than two months having symptoms; (5) being able to understand and perform all research data collection tasks; (6) having signed the informed consent. Exclusion criteria were: (1) previous surgery of carpal tunnel release on the examined extremity; (2) previous history of traumatic injury on the upper limb; (3) diagnosis of other musculoskeletal or neurological pathologies that may contribute to the development of CTS; (4) steroid or physiotherapy treatment in the last 6 months; (5) being pregnant.

### 2.4. Measures

Once patients were referred from Sector II of Aragon to the Faculty of Health Sciences by telephone, where two researchers (M.H.-S. and H.A.-B.) performed the measurements. Following verification that all selection criteria were met, all assessments were carried out in the same room at the Faculty of Health Sciences of the University of Zaragoza (Zaragoza, 50009).

#### 2.4.1. Sociodemographic Measures

Information on participant sex, age, body mass index, hand dominance, profession, physical activity, smoking, and alcohol habits were collected. Also, the bilaterality of symptoms, which hand would undergo surgery, current medication list, previous treatments for CTS, presence of comorbidities, date of onset of symptoms, and severity of CTS were reported.

All variables were collected from the patient’s medical records and verified during the examination session. Regarding CTS severity classification, data were taken from previous nerve conduction studies performed and described by the Surgery Unit. CTS severity was classified as mild, moderate, or severe, according to the criteria of the Hand and Wrist Surgery Unit, based on the results of the nerve conduction studies [18].

#### 2.4.2. Clinical Features

- Upper Limb Neurodynamic Test 1 (ULNT1)

**Figure 1:**
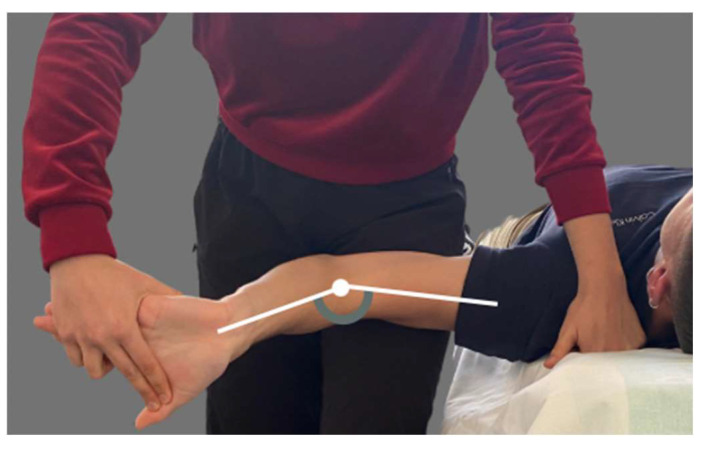
Upper Limb Tension Test 1.

Mechanosensitivity of the median nerve was assessed with ULNT1. An evaluator trained in neurodynamic tests and with previous experience in their application performed ULNT1. Moreover, a previous standardization of the ULNT1 was made. Participants were positioned supine with straight lower limbs. The procedure was carried out as described by Shacklock et al. [19]. The following sequence was performed: (1) 90° shoulder abduction; (2) shoulder external rotation; (3) forearm supination; (4) wrist and finger extension; (5) elbow extension (Figure 1). When symptoms appeared, movement was stopped, and structural differentiation was performed. Structural differentiation was performed by moving the region furthest away from the symptomatic area. Elbow range of motion, type of symptoms, symptom location and symptom intensity (0–10) were recorded for the test.

The range of movement of elbow extension was evaluated with a conventional goniometer (Jammar, Sammons Court, Bolingbrook, IL, USA). The degrees of movement were measured from 90° flexion to maximum elbow extension. The greater the degrees of elbow flexion, the more mechanosensitivity of the median nerve during the ULNT1.

Patients were instructed to report the type (Stretching, Pain, or Paresthesia) and localization (Fingers, Hand, Wrist, Forearm, Elbow, Arm, or Shoulder). Symptoms intensity was evaluated by a Numeric Pain Rating Scale from 0 to 10, being 0 “no symptoms” and 10 “maximum symptom experienced”.

Structural differentiation was considered positive if a movement of a distal body region that further loads or unloads the nervous system (e.g., finger flexion if the symptoms appear at the shoulder) without changing tension in adjacent structures such as muscles or tendons (sensitizing movements at a site distant to the symptoms) was indicative of neural involvement [11].

- Clinical outcomes

Symptoms, such as pain, paresthesia, and nocturnal symptoms, were evaluated with a specific Visual Analogue Scale (VAS) of 100 mm length, 0 being “no symptom” and 100 “maximum symptom experienced” for the last week. The Boston Carpal Tunnel Questionnaire (BCTQ) was used to assess the severity of symptoms and current function. It is divided into two sub-scales of symptoms, severity of symptoms is scored from 0 to 45, and function is scored from 0 to 55. The Spanish version is a valid and reliable instrument for CTS patients [20].

Hand grip was assessed with a hand dynamometer (JAMAR, Patterson Medical, Chicago, IL, USA), measured in kg. Patients were positioned, with the elbow supported at 90° of flexion and neutral pronation–supination. The patient was asked to squeeze the dynamometer as hard as possible, considering their pain tolerance, for seven seconds and three repetitions. The average of the maximal strength of the three measurements was registered as the final value [21].

The mechanical sensory threshold was measured in both hands at the midpoint of the distal phalanx of the five fingers. Semmes–Weinstein monofilaments (Touchtest, North Coast Medical Inc., Morgan Hill, CA, USA) were used starting from 2.83 (0.07 g) filament, which was considered normal. The tool has 16 monofilaments, of which 4 represent normal sensitivity (1.65 to 2.85) and 12 hypo-sensitivity (3.22 to 6.65). During monofilament testing, the patient was positioned supine, eyes closed, with the arm in a neutral position, palm upwards. Five stimuli were performed on each finger, of which at least 2 had to be felt. The sequence of the fingers for each stimulus was randomized to avoid patient learning. As soon as the patient identified two stimuli, the filament number was registered [22].

Quality of life was evaluated with the 36-item Short Form Survey (SF-36) and the Medical Outcomes Study Sleep Scale (MOS-sleep). The SF-36 measures eight items (physical function, role limitation physical, body pain, general health, energy, social functioning, role limitation, and emotional and mental health) and an additional item related to health evolution. It was scored from 0 to 100, with 100 being the best state of health. The Spanish version provides a comprehensive, efficient, and psychometrically sound method for measuring health [23].

The MOS Sleep Scale (MOSS-Sleep) survey is a 12-item self-report sleep measure that provides a concise assessment of important dimensions of sleep [24]. The scale yields a sleep problems index and six scale scores (sleep disturbance, snoring, awakening short of breath or with headache, sleep adequacy, somnolence, sleep problem index I and II). Higher scores indicate more sleep impairment, with 0 being the lowest score and 100 being the highest possible score for each item. The MOS-sleep survey demonstrates good psychometric properties and is sensitive to changes in patients with Neuropathic pain [25].

Finally, kinesiophobia was assessed by The Tampa Scale. The short form of the scale is composed of 11 questions scored from 1 to 4 each. Scores ranged from 11 points to 44 points. The higher the score, the higher the level of kinesiophobia. The scale has demonstrated good psychometric properties in chronic pain [26].

### 2.5. Statistical Analysis

Statistical analysis was performed using the Statistics Package for Social Science (SPSS v.25, IBM Inc., Armonk, NY, USA). Data normality was assessed by histograms and the Shapiro–Wilk test, with a significance threshold set at 0.05.

The mean and standard deviation were used to describe quantitative variables, while the median and maximum–minimum were calculated for the non-parametrical variables. Absolute frequencies and percentages were calculated for qualitative variables.

A Spearman rank-order correlation coefficient was calculated to analyze the associations of median nerve mechanosensitivity (elbow range of motion) with clinical features. The correlation coefficient was defined as 0–0.10 insignificant correlation, 0.11–0.39 weak correlation, 0.40–0.69 moderate correlation, 0.70–0.89 strong correlation, and 0.90–1.00 very strong correlation [27].

## 3. Results

### 3.1. Sociodemographic Characteristics

A cohort of forty-two patients (24 women and 18 men) with CTS included in the surgery waiting list of Healthcare Sector System II of Aragon (Zaragoza, Spain) were included. The flow diagram can be found in supplementary material (Supplementary Materials—Figure S1). Most patients suffered from one or more comorbidities (78.6%). The median of days between the first medical visit to the surgeon visit was 365.5 days (12 months). Sociodemographic variables are shown in Table 1 and clinical features in Supplementary Materials (Table S2).

CTS patients included in the surgical waiting list in Aragon have either severe (81%) or moderate severity (19%) levels of CTS. None of the patients had a mild severity.

### 3.2. Upper Limb Tension Test 1 Characteristics

**Figure 2:**
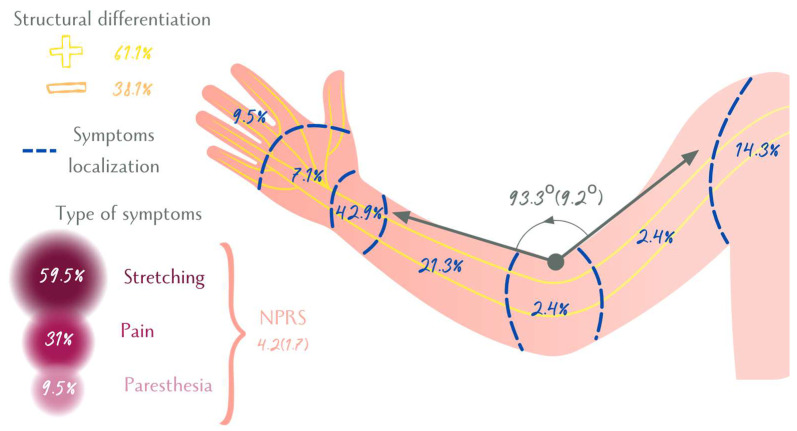
Upper Limb Tension Test 1 characteristics. Quantitative variables were presented as Mean (SD). NPRS: Numerical Pain Rating Scale.

When the mechanosensitivity of the median nerve was tested in pre-surgical CTS patients, symptoms typically appeared at approximately 90° of elbow flexion. Patients mainly localized their symptoms at the wrist (42.9%), forearm, and shoulder. In addition, the predominant symptom reported was stretching (59.5%). Finally, neural structural differentiation was positive for 59.5% of the sample. The characteristics of ULNT1 can be seen in Figure 2 and Supplementary Material (Table S3).

### 3.3. Association with the Mechanosensitivity of the Median Nerve

**Figure 3:**
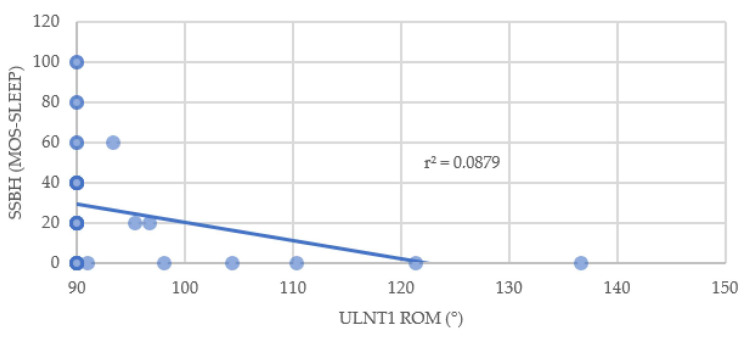
Relationship between elbow extension during ULNT1 and Awaken Short of Breath or with Headache. SSBH: Awaken Short of Breath or with Headache; ROM: Range of Movement; ULNT1: Upper Limb Tension Test 1.

**Figure 4:**
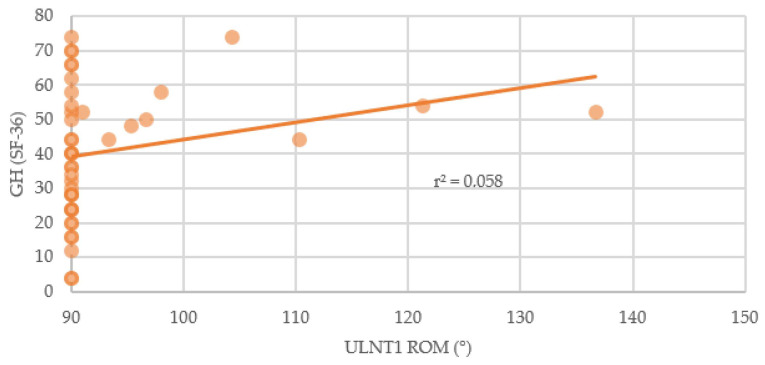
Relationship between elbow extension during ULNT1 and General Health. GH: General Health; ROM: Range of Movement; ULNT1: Upper Limb Tension Test 1.

**Figure 5:**
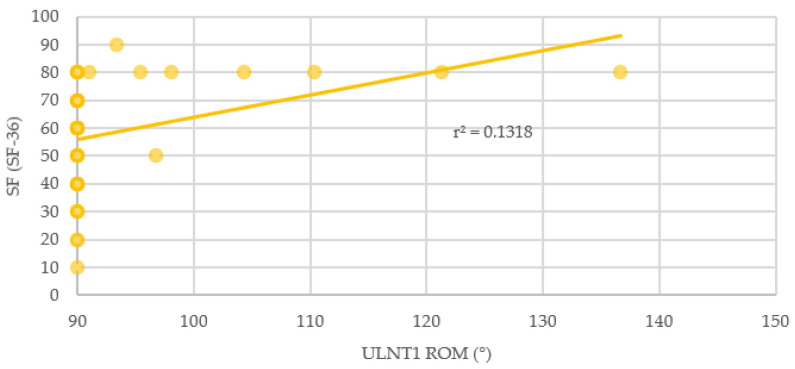
Relationship between elbow extension during ULNT1 and Social Functioning. SF: Social Functioning; ROM: Range of Movement; ULNT1: Upper Limb Tension Test 1.

Association of median nerve mechanosensitivity was found for the SF-36 questionnaire in the subscales General Health and Social Functioning. Also, the correlation between ULNT1 and the MOS Sleep Scale (Awaken Short of Breath or with Headache subscale) was significant (*p* < 0.05) (Table 2). Median nerve mechanosensitivity correlated weakly with Sleep short of headache/breath (Spearman’s rho = −0.353) (Figure 3) and General Health (Spearman’s rho = 0.367) (Figure 4) and moderately with Social Functioning (Spearman’s rho = 0.553) (Figure 5). No other correlation was found.

## 4. Discussion

This study examines the association between mechanosensitivity of the median nerve and clinical features in pre-surgical CTS patients, showing a low and moderate association between quality of sleep and life. The study was performed in a sample of pre-surgical CTS severe patients predominantly and with an average of 4 years with symptoms.

### 4.1. Upper Limb Tension Test 1 Characteristics

Jaberzadeh et al. [28] demonstrated that higher mechanosensitivity of the median nerve in the CTS group was evidenced by decreased elbow extension ROM. Along with this, increased elbow-flexor resistive torque and EMG activity of the biceps, flexor carpis radialis and triceps muscles, in response to the mechanical provocation of the median nerve at the ULNT1 test position. This could explain a predominant feeling of stretching at the onset of symptoms during the test.

Mechanosensitivity measured by ULNT1 for asymptomatic subjects is considered normal if symptoms appear between 130° and 150° of elbow extension in the forearm-to-fingers area according to Shacklock [19]. In our study, the median value was 90°. In addition, for many of the cases, it was impossible to start mobilizing the elbow due to the previous onset of symptoms during the ULNT1. In total, 81% of patients felt their symptoms in the area considered normal. Another study of patients with moderate CTS found that the mean range of movement was below the normal data [29]. In contrast, Jiménez-del-Barrio et al. [30] found similar results to our sample. The predominant symptom reported in their study was stretching, with a mean symptom intensity of 4/10. However, although the predominant location of the patients was at the level of the wrist and forearm, as in our study, our patients also reported symptoms above the elbow region. This may be due to the fact that the patients analyzed were more severe compared to the mild and moderate patients in Jiménez-del-Barrio et al.’s study [30].

Knowing that ULNT1 presents a high specificity (0.84) and moderate sensitivity (0.58) for the diagnosis of CTS [11], the different variables analyzed may provide a means of monitoring the progress of patients after intervention.

### 4.2. Association with the Mechanosensitivity of the Median Nerve

To our knowledge, the association between median nerve mechanosensitivity with symptoms, function, and psychosocial variables in CTS patients has not been studied.

In the present study, mechanosensitivity was found to correlate with Awaken Short of Breath or with Headache. In this case, those participants with greater median nerve mechanosensitivity had greater impairment of this aspect of sleep. Other studies have observed associations between sleep and clinical parameters in patients with CTS. Goorman et al. [31] determined that sleep disturbance and shorter sleep duration were both associated with lower hand function and higher pain. Patel et al. [32] observed that the greater the pain and the worse the function in patients with CTS, the poorer the sleep quality. Therefore, the quality of sleep should be considered an important factor when treating these patients.

Median nerve mechanosensitivity was also found to correlate with General Health. Those patients with a higher score in the section, less affectation, presented greater tolerance to mechanical stimulus on the median nerve. Furthermore, those patients with greater mechanosensitivity had a greater limitation of quality of life in the area of social function. Similarly, Khan et al. [33] observed similar correlations between symptoms and function with psychosocial factors in patients with CTS. The authors found that both pain, as measured by VAS, and function, as measured by the Boston questionnaire, correlated positively with anxiety and depression, which may impact a patient’s perception of general health and social function.

Although other clinical variables have not been associated with neurodynamic tests, it has been observed that women with CTS and a low educational level exhibited reduced grip and pinch strength and more catastrophic thinking [34]. In parallel, other psychosocial variables such as catastrophizing and kinesiophobia were found to be significantly associated with increased pain area in patients with carpal tunnel syndrome [35]. Finally, Nuñez-Cortés et al. demonstrated that a high level of physical activity was associated with a decrease in pain intensity and depressive symptoms in patients with CTS awaiting surgery [36]. Given this evidence, it is important to collect and evaluate information regarding the psychosocial aspects of patients with carpal tunnel syndrome.

Knowing that ULNT1 present a high specificity (0.84) and moderate sensitivity (0.58) for the diagnosis of CTS [11], the different variables analyzed can allow us to monitor the evolution of these patients after an intervention.

Limitations for this study include a small sample size, minimizing the bias due to the calculation of the sample size. The relatively small sample size in pathology such as CTS with high prevalence values may avoid the generalization of the results to populations in other geographical situations and with other sociodemographic characteristics. In addition, the sample was selected from a specific Spanish autonomous community. Therefore, the data found may not be extrapolated to other countries. These limitations could be mitigated using multicenter studies and with a larger sample size. Another limitation of the study was the measurement of the range of motion during the neurodynamic test. Only the range of motion of the elbow was measured, without quantifying the motion of the other joints positioned during the ULNT1. In some cases where mechanosensitivity was high, symptoms were reported while positioning the shoulder, forearm, wrist, and hand so that elbow flexion degrees of motion were not measurable. To avoid this limitation, a total measurement including the range of motion of all regions moved during ULNT1 should be included. Furthermore, due to the correlational nature of the study, causality cannot be inferred.

No previous study had evaluated mechanosensitivity in a sample of patients with severe CTS and with such a long period of symptoms. Furthermore, the mechanosensitivity of the median nerve had not been related to clinical variables in pre-surgical patients. Longer periods of pain or dysfunction are related to a higher degree of psychosocial discomfort [37]. Therefore, the current results may not be extrapolated to samples with a lower degree of severity and less time of evolution.

Finally, it should be noted that the lack of variability in elbow extension during ULNT1 can hinder the ability to demonstrate the relationship between clinical variables, leading to underestimation or misinterpretation of relationships between variables [38]. Therefore, future studies should be conducted with greater variability of results in neurodynamic tests.

## 5. Conclusions

An association between mechanosensitivity of the median nerve and quality of life and sleep was found. Psychosocial aspects should be taken into account when assessing pre-surgical patients with carpal tunnel syndrome.

## Figures and Tables

**Table 1:** Sociodemographic and medical history characteristics.

	Subjects (*n* = 42)
*Sex, n (%)*	
Female	24 (57.1)
Male	18 (42.9)
Age (Years) *	59.1 ± 12.7
BMI (kg/m^2^) *	29.3 ± 5.6
*Occupation, n (%)*	
Cleaning	13 (31.0)
Health Staff	6 (14.3)
Construction	5 (11.9)
Driver	3 (7.1)
Hostelry	3 (9.5)
Administration	5 (11.9)
Farming	5 (11.9)
Other	2 (4.8)
*Employment status, n (%)*	
Employed	24 (57.1)
Unemployed	4 (9.5)
Retired	11 (26.2)
Unable to work or disabled	3 (7.1)
*Physical activity (h/wk) ^†^*	0 (0–21)
*Domestic chores (h/wk) ^†^*	14 (0–56)
*Medication*	
No	8 (19.0)
One	14 (33.4)
Two or more	20 (47.6)
*Comorbidities, n (%)*	
None	9 (21.4)
Diabetes	6 (14.3)
Hypothyroidism	4 (9.5)
Hypertension	8 (19.0)
Obesity	12 (28.6)
Other	15 (35.7)
*Number of comorbidities, n (%)*	
None	9 (21.4)
One	13 (31.0)
Two	10 (23.8)
Three or more	10 (23.8)
*Alcohol consumption, n (%)*	
No	17 (40.5)
Monthly	7 (16.7)
Weekly	11 (26.2)
Daily	7 (16.7)
*Smoking status, n (%)*	
No	35 (83.3)
1–6 cigars per day	2 (4.8)
7–12 cigars per day	1 (2.4)
>20 cigars per day	4 (9.5)
*Bilateral symptoms, n (%)*	
Yes	36 (85.7)
No	6 (14.3)
*Severity, n (%)*	
Mild	0 (0)
Moderate	8 (19)
Severe	34 (81)
*Time with symptoms (years) ^†^*	4 (0.5–18)
*Previous treatments, n (%)*	
None	18 (42.9)
Pharmacology	11 (26.2)
Infiltration	2 (4.8)
Hand Splint	20 (47.6)
Physiotherapy	3 (7.1)
*Time from first medical visit to surgeon visit (d) ^†^*	365.5 (0–1921)

BMI: Body Mass Index. * Data are shown as Mean ± Standard Deviation. ^†^ Data are shown as Median (Minimum–Maximum).

**Table 2:** Association between mechanosensitivity of the median nerve (elbow extension) and clinical features.

		ULNT1 (°) *n* = 42
*Pain*	Spearman’s rho	–0.271
	*p*	0.082
*Nocturnal symptoms*	Spearman’s rho	–0.224
	*p*	0.154
*Paresthesia*	Spearman’s rho	–0.136
	*p*	0.390
*BCTQ*		
Symptoms	Spearman’s rho	–0.186
	*p*	0.236
Function	Spearman’s rho	–0.127
	*p*	0.423
*Grip strength*	Spearman’s rho	0.231
	*p*	0.141
*Mechanical sensory threshold*		
1st finger	Spearman’s rho	0.044
	*p*	0.780
2nd finger	Spearman’s rho	0.056
	*p*	0.726
3rd finger	Spearman’s rho	–0.070
	*p*	0.659
4th finger	Spearman’s rho	–0.203
	*p*	0.197
5th finger	Spearman’s rho	–0.053
	*p*	0.737
*SF-36*		
Physical Function	Spearman’s rho	0.121
	*p*	0.445
Role limitation Physical	Spearman’s rho	0.186
	*p*	0.239
Body Pain	Spearman’s rho	0.166
	*p*	0.294
General Health	Spearman’s rho	0.367
	*p*	0.017 *
Energy	Spearman’s rho	0.245
	*p*	0.117
Social Functioning	Spearman’s rho	0.553
	*p*	<0.001 *
Role limitation emotional	Spearman’s rho	0.297
	*p*	0.056
Mental Health	Spearman’s rho	0.196
	*p*	0.213
Health evolution	Spearman’s rho	0.069
	*p*	0.069
*MOS-sleep*		
Sleep Disturbance	Spearman’s rho	0.002
	*p*	0.991
Snoring	Spearman’s rho	0.096
	*p*	0.547
Awaken Short of Breath or with Headache	Spearman’s rho	−0.353
	*p*	0.022 *
Sleep adequacy	Spearman’s rho	0.083
	*p*	0.602
Somnolence	Spearman’s rho	−0.026
	*p*	0.872
Sleep problems I	Spearman’s rho	−0.192
	*p*	0.224
Sleep problems II	Spearman’s rho	−0.151
	*p*	0.340
*Tampa scale*	Spearman’s rho	−0.168
	*p*	0.289

BCTQ: Boston Carpal Tunnel Questionnaire; MOS-sleep: Medical Outcomes Study Sleep Scale; SF-36: 36-Item Short Form Survey; ULNT1: Upper Limb Nerve Test 1. * *p* value < 0.05.

## Data Availability

The original contributions presented in the study are included in the article/Supplementary Material, further inquiries can be directed to the corresponding author.

## Supplementary Materials

The following supporting information can be downloaded at: https://www.mdpi.com/article/10.3390/brainsci14060615/s1, Table S1: STROBE Statement; Table S2: Clinical features; Table S3: Upper Limb Nerve Test 1 Characteristics; Figure S1: STROBE Flow diagram. Ref. [39] is cited in Supplementary Materials.

## References

[1] Wipperman J., Goerl K. (2016). Carpal Tunnel Syndrome: Diagnosis and Management. Am. Fam. Physician.

[2] Padua L., Cuccagna C., Giovannini S., Coraci D., Pelosi L., Loreti C., Bernabei R., Hobson-Webb L.D. (2023). Carpal tunnel syndrome: Updated evidence and new questions. Lancet Neurol..

[3] Burton C.L., Chen Y., Chesterton L.S., Van Der Windt D.A. (2018). Trends in the prevalence, incidence and surgical management of carpal tunnel syndrome between 1993 and 2013: An observational analysis of UK primary care records. BMJ Open.

[4] Steimle J., Gabriel S., Tarr R., Kohrs B., Johnston P., Martineau D. (2020). Comparing Diagnostic and Treatment Recommendations of Carpal Tunnel Syndrome Available on the Internet With AAOS Clinical Practice Guidelines. Hand.

[5] Bourke H.E., Read J., Kampa R., Hearnden A., Davey P.A. (2011). Clinic-based nerve conduction studies reduce time to surgery and are cost effective: A comparison with formal electrophysiological testing. Ann. R. Coll. Surg. Engl..

[6] Pimentel B.F.R., Faloppa F., Tamaoki M.J.S., Belloti J.C. (2018). Effectiveness of ultrasonography and nerve conduction studies in the diagnosing of carpal tunnel syndrome: Clinical trial on accuracy. BMC Musculoskelet. Disord..

[7] Sartorio F., Dal Negro F., Bravini E., Ferriero G., Corna S., Invernizzi M., Vercelli S. (2020). Relationship between nerve conduction studies and the Functional Dexterity Test in workers with carpal tunnel syndrome. BMC Musculoskelet. Disord..

[8] Sonoo M., Menkes D.L., Bland J.D.P., Burke D. (2018). Nerve conduction studies and EMG in carpal tunnel syndrome: Do they add value?. Clin. Neurophysiol. Pract..

[9] Erickson M., Lawrence M., Stegink Jansen C., Coker D., Amadio P., Cleary C. (2019). Carpal Tunnel Syndrome: A Summary of Clinical Practice Guideline Recommendations—Using the Evidence to Guide Physical Therapist Practice. J. Orthop. Sports Phys. Ther..

[10] Ellis R., Carta G., Andrade R.J., Coppieters M.W. (2022). Neurodynamics: Is tension contentious?. J. Man. Manip. Ther..

[11] Bueno-Gracia E., Tricás-Moreno J.M., Fanlo-Mazas P., Malo-Urriés M., Haddad-Garay M., Estébanez-de-Miguel E., Hidalgo-García C., Krauss J.R. (2016). Validity of the Upper Limb Neurodynamic Test 1 for the diagnosis of Carpal Tunnel Syndrome. The role of structural differentiation. Man. Ther..

[12] Trillos M.C., Soto F., Briceno-Ayala L. (2018). Upper limb neurodynamic test 1 in patients with clinical diagnosis of carpal tunnel syndrome: A diagnostic accuracy study. J. Hand Ther..

[13] Vanti C., Bonfiglioli R., Calabrese M., Marinelli F., Guccione A., Violante F.S., Pillastrini P. (2011). Upper Limb Neurodynamic Test 1 and symptoms reproduction in carpal tunnel syndrome. A validity study. Man. Ther..

[14] Vanti C., Conteddu L., Guccione A., Morsillo F., Parazza S., Viti C., Pillastrini P. (2010). The Upper Limb Neurodynamic Test 1: Intra- and Intertester Reliability and the Effect of Several Repetitions on Pain and Resistance. J. Manip. Physiol. Ther..

[15] Baselgia L.T., Bennett D.L., Silbiger R.M., Schmid A.B. (2017). Negative Neurodynamic Tests Do Not Exclude Neural Dysfunction in Patients With Entrapment Neuropathies. Arch. Phys. Med. Rehabil..

[16] Atroshi I., Gummesson C., Johnsson R., Ornstein E., Ranstam J., Rosén I. (1999). Prevalence of carpal tunnel syndrome in a General Population. JAMA.

[17] Soto-Alvarez J. (1995). Importancia del tamaño de la muestra en la investigación clínica. Rev. Clin. Esp..

[18] Bland M. (2000). Producing benchmarks for clinical practice. Prof. Nurse.

[19] Shacklock M.O. (2005). Clinical Neurodynamics.

[20] Oteo-Álvaro Á., Marín M.T., Matas J.A., Vaquero J. (2016). Validación al castellano de la escala Boston Carpal Tunnel Questionnaire. Med. Clin..

[21] Trampisch U.S., Franke J., Jedamzik N., Hinrichs T., Platen P. (2012). Optimal jamar dynamometer handle position to assess maximal isometric hand grip strength in epidemiological studies. J. Hand Surg..

[22] Fonseca M.D.C.R., Elui V.M.C., Lalone E., Da Silva N.C., Barbosa R.I., Marcolino A.M., Ricci F.P.F.M., MacDermid J.C. (2018). Functional, motor, and sensory assessment instruments upon nerve repair in adult hands: Systematic review of psychometric properties. Syst. Rev..

[23] Alonso J., Prieto L., Anto J.M. (1995). La versión española del SF-36 Health Survey (Cuestionario de Salud SF-36): un instrumento para la medida de los resultados clínicos. Med. Clin..

[24] Spritzer K.L., Hays R.D. (2003). MOS Sleep Scale: A Manual for Use and Scoring.

[25] Rejas J., Ribera M., Ruíz M., Masramon X. (2005). Psychometric Properties of the Mos-Sleep Scale in Neuropathic Pain (Nep) Syndromes. Value Health.

[26] French D.J., France C.R., Vigneau F., French J.A., Evans R.T. (2007). Fear of movement/(re)injury in chronic pain: A psychometric assessment of the original English version of the Tampa scale for kinesiophobia (TSK). Pain.

[27] Schober P., Schwarte L.A. (2018). Correlation Coefficients: Appropriate Use and Interpretation. Anesth. Analg..

[28] Jaberzadeh S., Zoghi M. (2013). Mechanosensitivity of the median nerve in patients with chronic carpal tunnel syndrome. J. Bodyw. Mov. Ther..

[29] Jiménez-del-Barrio S. (2016). Efectos del tratamiento fisioterápico mediante Fibrolisis Diacutánea en pacientes con Síndrome del Túnel Carpiano.

[30] Jiménez Del Barrio S., Ceballos-Laita L., Bueno-Gracia E., Rodríguez-Marco S., Haddad-Garay M., Estébanez-De-Miguel E. (2021). Effects of Diacutaneous Fibrolysis on Mechanosensitivity, Disability, and Nerve Conduction Studies in Mild to Moderate Carpal Tunnel Syndrome: Secondary Analysis of a Randomized Controlled Trial. Phys. Ther..

[31] Goorman A.M., Dawson S., Schneck C., Pierce D. (2019). Association of Sleep and Hand Function in People With Carpal Tunnel Syndrome. Am. J. Occup. Ther..

[32] Patel A., Culbertson M.D., Patel A., Hashem J., Jacob J., Edelstein D., Choueka J. (2014). The Negative Effect of Carpal Tunnel Syndrome on Sleep Quality. Sleep Disord..

[33] Khan F., Shehna A., Ramesh S., Sandhya K., Paul R. (2017). Subjective symptoms of carpal tunnel syndrome correlate more with psychological factors than electrophysiological severity. Ann. Indian Acad. Neurol..

[34] Núñez-Cortés R., Cruz-Montecinos C., Antúnez-Riveros M.A., Pérez-Alenda S. (2020). Does the educational level of women influence hand grip and pinch strength in carpal tunnel syndrome?. Med. Hypotheses.

[35] Núñez-Cortés R., Carrasco J.J., Salazar-Méndez J., Torreblanca-Vargas S., Pérez-Alenda S., Calatayud J., Lluch E., Horment-Lara G., Cruz-Montecinos C., Cerda M. (2024). Psychological factors are associated with pain extent in patients with carpal tunnel syndrome. Physiother. Theory Pract..

[36] Núñez-Cortés R., Cruz-Montecinos C., Torreblanca-Vargas S., Andersen L.L., Tapia C., Ortega-Palavecinos M., López-Bueno R., Calatayud J., Pérez-Alenda S. (2023). Social determinants of health and physical activity are related to pain intensity and mental health in patients with carpal tunnel syndrome. Musculoskelet. Sci. Pract..

[37] Tagliaferri S.D., Fitzgibbon B.M., Owen P.J., Miller C.T., Bowe S.J., Belavy D.L. (2022). Brain structure, psychosocial, and physical health in acute and chronic back pain: A UK Biobank study. Pain.

[38] Asamoah M.K. (2014). Re-examination of the limitations associated with correlational research. J. Edu Res. Rev..

[39] Vandenbroucke J.P., von Elm E., Altman D.G., Gøtzsche P.C., Mulrow C.D., Pocock S.J., Poole C., Schlesselman J.J., Egger M. (2014). STROBE Initiative. Strengthening the Reporting of Observational Studies in Epidemiology (STROBE): Explanation and elaboration. Int. J. Surg..

