# Supplementary material for: Understanding the Mechanosensitivity of the Median Nerve in Pre-Surgical Carpal Tunnel Syndrome Patients: A Correlational Study
Source: Brain Sci. 2024 Jun 19;14(6):615. doi: 10.3390/brainsci14060615 (PMC11201705; doi:10.3390/brainsci14060615)
Supplement: Supplementary file 1 [file brainsci-14-00615-s001.zip › brainsci-3065931-supplementary.pdf]

Table S1: STROBE Statement—Checklist of items that should be included in reports of cross-sectional studies

	Item No	Recommendation	Line (page)
**Title and abstract**	1	(*a*) Indicate the study's design with a commonly used term in the title or the abstract	1-4 (1)
		(*b*) Provide in the abstract an informative and balanced summary of what was done and what was found	18-32 (1)
**Introduction**			
Background/rationale	2	Explain the scientific background and rationale for the investigation being reported	36-54 (1-2)
Objectives	3	State specific objectives, including any prespecified hypotheses	55-57 (2)
**Methods**			
Study design	4	Present key elements of study design early in the paper	60-68 (2)
Setting	5	Describe the setting, locations, and relevant dates, including periods of recruitment, exposure, follow-up, and data collection	60-68 78-76 (2)
Participants	6	(*a*) Give the eligibility criteria, and the sources and methods of selection of participants	85-92 (2)
Variables	7	Clearly define all outcomes, exposures, predictors, potential confounders, and effect modifiers. Give diagnostic criteria, if applicable	94-177 (3-5)
Data sources/ measurement	8*	For each variable of interest, give sources of data and details of methods of assessment (measurement). Describe comparability of assessment methods if there is more than one group	94-177 (3-5)
Bias	9	Describe any efforts to address potential sources of bias	94-177 (3-5)
Study size	10	Explain how the study size was arrived at	70-76 (2)
Quantitative variables	11	Explain how quantitative variables were handled in the analyses. If applicable, describe which groupings were chosen and why	X
Statistical methods	12	(*a*) Describe all statistical methods, including those used to control for confounding	179-190 (5)
		(*b*) Describe any methods used to examine subgroups and interactions	
		(*c*) Explain how missing data were addressed	
		(*d*) If applicable, describe analytical methods taking account of sampling strategy	
		(*e*) Describe any sensitivity analyses	
**Results**			
Participants	13	(a) Report numbers of individuals at each stage of study—eg numbers potentially eligible, examined for eligibility, confirmed eligible, included in the study, completing follow-up, and analysed	193-196 (5)

		(b) Give reasons for non-participation at each stage	X
		(c) Consider use of a flow diagram	Fig S2
Descriptive data	14	(a) Give characteristics of study participants (eg demographic, clinical, social) and information on exposures and potential confounders	Table 1 (5-6)
		(b) Indicate number of participants with missing data for each variable of interest	Fig S2
Outcome data	15	Report numbers of outcome events or summary measures	Table 1; S1; S2. Figure 2 (5-7)
Main results	16	(*a*) Give unadjusted estimates and, if applicable, confounder-adjusted estimates and their precision (eg, 95% confidence interval). Make clear which confounders were adjusted for and why they were included	Table 2, Figure 3-5 (7-9)
		(*b*) Report category boundaries when continuous variables were categorized	
		(*c*) If relevant, consider translating estimates of relative risk into absolute risk for a meaningful time period	
Other analyses	17	Report other analyses done—eg analyses of subgroups and interactions, and sensitivity analyses	X
**Discussion**			
Key results	18	Summarise key results with reference to study objectives	239-241 (9)
Limitations	19	Discuss limitations of the study, taking into account sources of potential bias or imprecision. Discuss both direction and magnitude of any potential bias	278-288 (10)
Interpretation	20	Give a cautious overall interpretation of results considering objectives, limitations, multiplicity of analyses, results from similar studies, and other relevant evidence	243-277 (9-10)
Generalisability	21	Discuss the generalisability (external validity) of the study results	243-277 (9-10)
**Other information**			
Funding	22	Give the source of funding and the role of the funders for the present study and, if applicable, for the original study on which the present article is based	305-306 (11)

Figure S1: STROBE Flow diagram

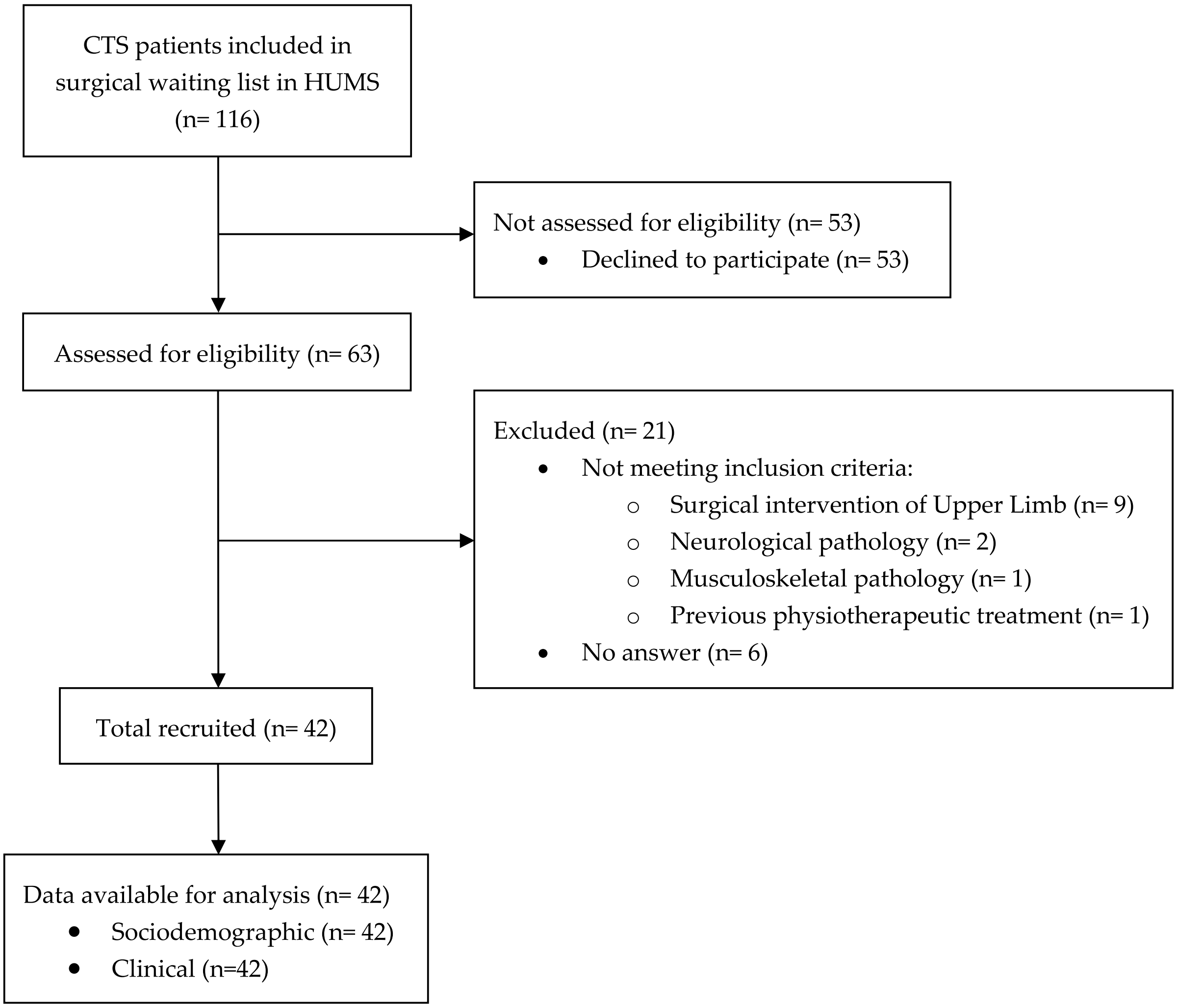

(Vandenbroucke JP, von Elm E, Altman DG, Gøtzsche PC, Mulrow CD, Pocock SJ, Poole C, Schlesselman JJ, Egger M; STROBE Initiative. Strengthening the Reporting of Observational Studies in Epidemiology (STROBE): explanation and elaboration. Int J Surg. 2014 Dec;12(12):1500-24.)

Table S2. Clinical features.

	Subjects (n=42)
Pain (mm)	50 (0-87)
Nocturnal symptoms (mm)	67 (3-100)
Paresthesia (mm)	73 (35-100)
BCTQ	
Symptoms (0-45)	19 (7-31)
Function (0-55)	32 (13-54)
Grip strength (kg)	19.5 (6-46)
Mechanical sensory threshold (1.65-6.65)	
1st finger	3.22 (2.36-6.65)
2nd finger	3.61 (2.36-6.65)
3rd finger	3.22 (1.65-6.65)
4th finger	3.22 (1.65-6.65)
5th finger	3.22 (2.36-6.65)
SF-36	
Physical Function (0-100)	46.7 (0-66.7)
Role limitation Physical (0-100)	0 (0-50)
Body Pain (0-100)	26.7 (0-60)
General Health (0-100)	40 (4-73.6)
Energy (0-100)	54.2 (8.3-75)
Social Functioning (0-100)	60 (10-90)
Role limitation emotional (0-100)	33.3 (0-100)
Mental Health (0-100)	66.7 (10-83.3)
Health evolution (0-100)	25 (0-84)
MOS-sleep	
Sleep Disturbance (0-100)	26.5 (0-70)
Snoring (0-100)	40 (0-100)
Sleep short of headache/breath (0-100)	20 (0-100)
Sleep adequacy (0-100)	50 (10-90)
Sleep Somnolence (0-100)	33.3 (0-60)
Sleep problems I (0-100)	36.7 (3.3-66.7)
Sleep problems II (0-100)	36.1 (8.9-66.7)
Tampa scale (11-44)	27 (16-44)

Data is shown as Median (Minimum-Maximum). BCTQ: Boston Carpal Tunnel Questionnaire; MOS-sleep: Medical Outcomes Study Sleep Scale; ROM: Range of Movement; SF-36: 36-Item Short Form Survey; ULNT1: Upper Limb Nerve Test 1

Table S3. Upper Limb Nerve Test 1 Characteristics.

ULNT1	**Subjects (n=42)**
Elbow ROM (°)	93.3±9.2 (90-136.67)
Intensity of sensation (0-10)	4.2±1.7 (1-7.3)
Localization, n (%)	
Fingers	4 (9.5)
Hand	3 (7.1)
Wrist	18 (42.9)
Forearm	9 (21.4)
Elbow	1 (2.4)
Arm	1 (2.4)
Shoulder	6 (14.3)
Structural differentiation, n (%)	
Positive	25 (59.5)
Negative	16 (38.1)
Sensation, n (%)	
Stretching	25 (59.5)
Pain	13 (31.0)
Paresthesia	4 (9.5)
Other	0 (0)

ROM: Range of motion.
